# Acquired Visual Deficits Independent of Lesion Site in Acute Stroke

**DOI:** 10.3389/fneur.2020.00705

**Published:** 2020-07-17

**Authors:** Chamini Wijesundera, Algis J. Vingrys, Tissa Wijeratne, Sheila G. Crewther

**Affiliations:** ^1^School of Psychology and Public Health, La Trobe University, Melbourne, VIC, Australia; ^2^Department of Neurology, Sunshine Hospital, The University of Melbourne, Parkville, VIC, Australia; ^3^Department of Optometry and Vision Sciences, The University of Melbourne, Parkville, VIC, Australia

**Keywords:** visual function, acute stroke, visual field, visual acuity-in-noise, ischemic, vision, Melbourne Rapid Field-Neural (MRFn)

## Abstract

Most clinical diagnoses of stroke are based on the persistence of symptoms relating to consciousness, language, visual-field loss, extraocular movement, neglect (visual), motor strength, and sensory loss following acute cerebral infarction. Yet despite the fact that most motor actions and cognition are driven by vision, functional vision *per se* is seldom tested rigorously during hospitalization. Hence we set out to determine the effects of acute stroke on functional vision, using an iPad application (Melbourne Rapid Field-Neural) that can be used to assess vision (visual acuity and visual field sensitivity) at the bedside or in the emergency ward in about 6 min per eye. Our convenience sample comprised 60 (29–88 years, 65 ± 14 years, 33 males) of 160 sequentially presenting first episode, acute (<7 days) ischemic stroke patients at Sunshine Hospital, Melbourne. One hundred patients were excluded due to existing eye disease, inadequate radiological confirmation, inability to comply with English directions or too ill to participate. Stroke cases were compared with 37 (29–85 years, 64 ± 12 years,14 males) similar-aged controls using a Mann-Whitney *U*-test. A significant loss in visual field sensitivity was measured in 68% of stroke cases (41/60, Mean Deviation: Stroke: −5.39 ± 6.26 dB, Control: 0.30 ± 0.60 dB, MWU = 246, *p* < 0.0001). Surprisingly, 44% (18/41) of these patients were unaware of their field loss. Although high contrast visual acuity was unaffected in most (55/60) patients, visual acuity-in-noise was reduced in 62% (37/60, Stroke: mean 6/12−2, log MAR 0.34 ± 0.21 vs. Control: mean 6/7·5–2, log MAR 0.14 ± 0.10; MWU = 470, *p* < 0.0001). Visual field defects were associated with all occipital, parietal and posterior cerebellar artery strokes while 9/15 middle cerebral artery lesions and 11 lesions in other brain regions were also associated with visual field defects. Our findings demonstrate that ~2/3 of acute first episode ischemic stroke patients experience acquired vision deficits, often unrelated to the confirmed lesion site. Our results also imply that visual dysfunction may be associated with a more generalized cerebral dysfunction while highlighting the need for bedside testing of vision for every stroke patient and demonstrating the translational clinical value of the “Melbourne Rapid Field- Neural” iPad application.

**Clinical Trial:** http://www.ANZCTR.org.au/ACTRN12618001111268.aspx.

## Introduction

Stroke is categorized by the World Health Organization as rapidly developing clinical signs of focal cerebral dysfunction due to vascular compromise, lasting more than 24 h, or leading to death (1, 2). Stroke is the leading cause of adult disability and the second leading cause of death worldwide (3). The American Stroke Association guidelines for the early management of acute ischemic stroke assessment emphasize testing of the level of consciousness, motor strength, items relating to confrontation visual field measurements, horizontal eye movements and visual inattention (4). However, visual function *per se* is seldom examined rigorously in the emergency room or during initial hospitalization for stroke (5), despite the central role vision plays in driving most human brain functions such as eye movements (6), attention (7), cognition (8), emotional responses (9), motor actions (6), and occupying larger volumes of cortical and subcortical regions in the human brain than do motor functions (10, 11).

Previous studies have reported that ~92% of the 915 stroke patients (5), who were referred to hospital eye clinics in the UK within a median of 22 days and up to 3 months post-stroke, have been reported as having some form of a visual deficit (12) with post chiasmal lesions in the lateral geniculate body (1%) (13), optic tract (6%), in the optic radiations (33%), and occipital lobes (54%). The commonest persistent visual deficits included visual field loss (hemianopia, quadrantanopia) (5), perceptual (visual inattention/neglect) (14) and eye movement disorders (5). Unfortunately, the recruitment criteria for the study of Rowe and colleagues (5) did not mention the number of unselected patients screened, nor the number with pre-existing eye diseases that may have confounded the effects of acute stroke on vision.

Ptosis has also been identified as a common indicator of transient ischemic attacks and midbrain infarctions (15) while impaired saccades, smooth pursuits (16), and nystagmus are reported to be more prevalent following frontal lobe, cerebellar and brainstem infarctions (17). Other stroke related visual anomalies have also been reported to be under diagnosed as ocular misalignment and gaze deficits can be subtle and patients are often unaware or asymptomatic for these changes (18, 19), with two-thirds of patients showing unilateral visual neglect following acute right hemisphere parietal stroke (20). Furthermore, the application of a battery of three bedside oculomotor tests (HINTS) measuring head impulse, nystagmus, and test of skew have proven accurate and reliable for the identification of acute stroke following acute vertigo presentations (21).

Indeed an acute stroke test battery (4) measuring distance visual acuity in each eye (22, 23), visual neglect (20), and ocular misalignment has been proposed recently (24). The battery includes tests for diplopia, pupil dysfunction, nystagmus and eye movement deficiency as well as more subtle tropia, phoria, and extraocular motor function in the cardinal positions of gaze, given that the cranial nerves III, IV and VI are supplied by a myriad of arteriole blood vessels on the same side as the eye such that they are susceptible to ocular motor dysfunction in ischemic conditions (24). However, the battery is not yet established as a regular neurological routine and most current bedside visual field assessments are performed using hand/finger confrontation (25) even though this method has been reported as having limited value for the detection of visual field loss (26, 27).

Confrontation continues to be used for bedside screening of stroke patients due to the difficulty of applying commercial visual field devices that require a degree of patient mobility and head/face coordination for testing (28). As a consequence, the nature of acquired visual field deficit in the acute phase of stroke (<72 h) has not been evaluated rigorously to date, though the advent of modern technology, and in particular tablet devices, afford ideal interfaces and test platforms for the testing of vision in hospitalized patients by their bedside (26, 29). A newly developed iPad tablet application for measuring visual field integrity known as Melbourne Rapid Field-Neural (MRFn) has recently been validated against the gold standard Humphrey Visual Field Analyzer (30) making it an useful tool to measure the integrity of functional vision across the visual fields of both eyes in hospitalized patients. The MRFn app also comes with the ability to test high contrast visual acuity with a Landolt C and visual acuity in noise (i.e., visual stimulus is embedded in a background of white noise) aimed at measuring threshold perception following the decomposition of the contrast of the target (30, 31). Therefore, measuring visual acuity performance in background noise provides useful insights into the neural mechanisms and computations needed to solve visual recognition (32–34) as demonstrated in the psychophysical testing of neurotypical and psychiatry patients with major depressive disorder (35).

Thus, the aim of this study was to utilize the MRFn (Melbourne Rapid Field-Neural) iPad application to measure visual acuity with high contrast targets, visual acuity-in-noise and visual field integrity in first episode hospitalized ischemic acute stroke patients with no prior history of ocular disorder. We hypothesize a decrement in vision post stroke acutely.

## Materials and Methods

The clinical ethics has been approved by the local review board (Western Health Ethics Committee HREC/16/WH/1) and was conducted in accordance with the tenets of the Declaration of Helsinki with all participants (or their carers) providing informed consent.

### Participants

**Figure 1:**
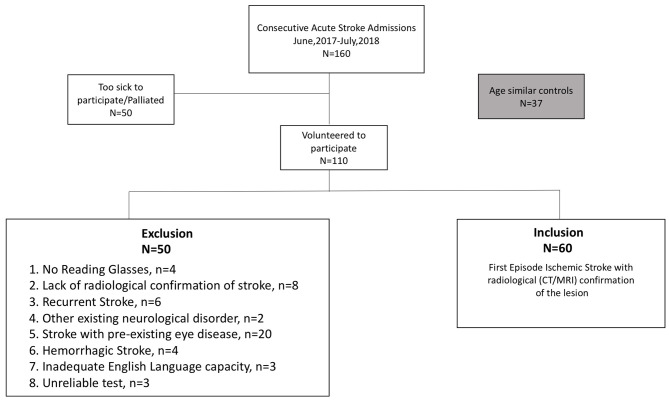
Consort diagram (36).

Our convenience sample of cases comprised 160 sequentially presenting, stroke patients (29–95 years, 68 ± 14.5years, 89 males) admitted to Sunshine Hospital, Melbourne, between June 2017 and July 2018. Patients were invited to volunteer for a subjective assessment of vision (visual acuity [high contrast and in noise] and visual fields) and those who agreed and, who met our inclusion criteria (i.e., first episode ischemic stroke with radiological confirmation, the availability of current habitual reading glasses) (Figure 1) were tested while wearing their habitual reading spectacles at their bedside using the Melbourne Rapid Field-Neural (MRFn) application. Refractions were not performed at the hospital rather their verbal history was used to determine the adequacy of current reading glasses. All testing was performed during the first week (usually day 2 or day 3) of hospital stay. Sixty first episode acute ischemic stroke patients (29–88 years, 65 ± 14 years, 33 males) met our inclusion criteria and had their data analyzed for this study. One hundred patients (63%) were excluded from analysis for the exclusion criteria shown in Figure 1.

Thirty-seven age-similar healthy controls (29–85 years, 64 ± 12 years,14 males) were recruited following a comprehensive routine eye examination at an optometry practice of one of the authors (CW) after providing informed written consent for participation. These participants showed no evidence of current or past ocular and neurological disorders and were wearing their habitual reading glasses.

Stroke diagnosis and localization of the vascular source of the lesion was determined at the time of admission by a neurologist with routine Computed Tomography (CT) or Magnetic Resonance Imaging (MRI). The greater spatial resolution of MRI was utilized to identify small volume ischemic changes often associated with minor strokes (37). This information was used to confirm the diagnosis and facilitate a structure-function analysis with the visual capacity (38).

### Melbourne Rapid Field-Neural iPad (MRFn) Application

**Figure 2:**
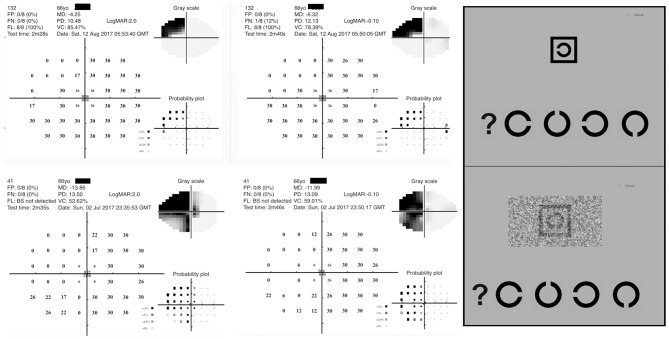
Diagnostic features of the Melbourne Rapid Field Neural (MRFn) test. **(Left)** Visual field outcomes for two acute stroke patients. **(Right)** Screen capture of the acuity optotypes used in the test (high contrast acuity-top, visual acuity-in-noise-bottom) with the response options at the bottom of the each presented optotypes.

The Melbourne Rapid Field application (GLANCE Optical Pty Ltd, Melbourne, Australia) measures visual acuity and visual field thresholds across the central visual field using an iPad tablet (12.9 inches iPad Pro) (39). Stroke cases sat on the hospital bed or on a bedside chair during the testing whereas controls performed the test on a bench in a clinical optometry practice at 33–38 cm working distance. The visual field test pattern used by MRFn is a reduced 24-2 Humphrey Field Analyser (HFA) test grid with 4 extra spots added to the fovea (Figure 2) (30). Spot size scaling results in a fixed threshold of 30 dB (Figure 2) at all locations (30). Previous studies find the MRFn returns outcomes that are strongly correlated to HFA thresholds on both a global and regional basis (40, 41).

In visual field testing, patients were required to respond to the presence of a spot by either tapping the screen or the spacebar of the iPad keyboard. All chose to tap the keyboard space bar indicating adequate manual control. We found one patient with a frontal lobe lesion who had difficulty tapping the space bar and preferred to tap the screen directly to complete testing. There were two other subjects who adopted their non-dominant hand for motor tasks after the stroke and used it for the visual assessment, all other participants used their dominant hand. Reliability (false positive, false negative and fixation loss) was routinely polled during testing.

The visual acuity test presents a high contrast “Landolt C” target (Figure 2) on a bright background (130 cd/sq.m) as well as the same “Landolt C” target embedded in luminance noise, generated using a psychometric model accounting for true acuity and noise in the visual system, with the reduction of the contrast sensitivity of the background spatial vision by 10% of the high contrast “Landolt C” optotype (42). Visual acuity-in noise has not been previously tested in acute stroke, but given the past reports for abnormality of noise-related tasks in acquired neurological disorders and in stroke cases well-after the onset of stroke (43, 44), we tested our acute stroke cohort expecting some may have difficulty recognizing visual targets immersed in noise.

### Testing Procedures

Visual acuity and the visual fields of both eyes of all study participants were measured monocularly in ambient hospital room lighting. The lighting has been found to have little impact on test outcomes (45) provided reflections off the screen are avoided. Screen brightness was set to maximum for 10 min prior to testing, to stabilize luminous output (46). Verbal instruction on test performance was given at the bedside and patients were allowed a practice trial before starting the test.

As most participants were naive to tablet perimetry, the preferred eye was tested first with operator feedback for training and learning of how to do the test. This eye was retested after the training phase before testing the fellow eye.

### Data Analysis

Comparisons between stroke and control groups were made for visual acuity, visual acuity-in-noise, and the mean deviation (MD) of the visual field. The mean deviation is determined from a pointwise comparison of contrast thresholds (dB) to age-related normals provided by the MRFn App. The time taken to complete vision assessments was also recorded.

Although both eyes were tested, the eye ipsilateral to the CT/MRI defined lesion was analyzed in the stroke group and compared to the RE (Right Eye) of controls (comparison to the fellow eye does not change our findings).

**Figure 3:**
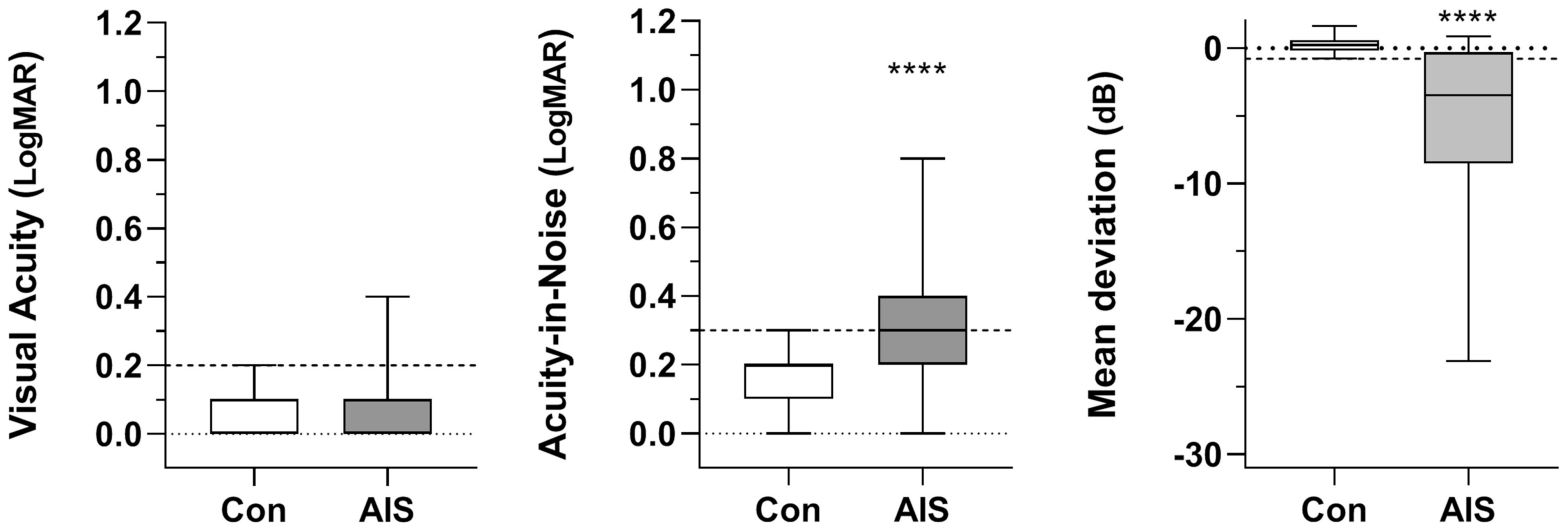
Box-and-whisker plots for visual acuity, visual acuity-in-noise and the Mean Deviation of the visual field for the control (Con) and acute ischemic stroke (AIS) groups. Horizontal dashed lines in each panel show 99th percentile for control data. Significant differences between groups (MWU, *P* < 0.0001) have been identified with asterisks (_****_).

Non-parametric statistics (Mann–Whitney *U*-tests) were employed given the heterogeneity and variability of data in the stroke group (Figure 3). All group data are shown as box-and-whisker plots, with whiskers identifying the total range of the data set. The 99th percentile of controls was used as the criterion to identify “abnormal” outcomes. Levene's test was used to compare group variances. Statistical analysis was conducted using GraphPad Prism v7.00 for Windows www.graphpad.com.

## Results

Of the 160 stroke presentations (Figure 1) MRFn testing could be performed and was successfully completed in 108 (68%) patients. Of these, 48 cases did not meet our inclusion criteria (first episode ischemic stroke with radiological confirmation, Figure 1) leaving 60 cases of acute ischemic stroke for analysis. First episode acute stroke patients were able to perform the tests accurately at their bedside, in under 5.4 ± 0.8 min per eye. Control patients completed all tests in under 4.0 ± 0.3 min per eye.

High contrast visual acuity (VA) was not significantly affected by acute ischemic stroke (Figure 3). Only 5 patients (8%) showed a one-line reduction in VA (mean 6/7·5+1, log MAR 0.09 ± 0.10) relative to controls (mean 6/6-3, log MAR 0.07 ± 0.06: MWU = 1,045, *p* = 0.30).

Statistically significant deterioration was found in the visual acuity-in-noise of 37/60 stroke cases (62%) despite having normal high contrast acuity (Stroke: mean 6/12-2, log MAR 0.34 ± 0.21 vs. Control: mean 6/7·5-2, log MAR 0.14 ± 0.10; MWU = 470, *p* < 0.0001). The stroke group also showed much larger variability in acuity-in-noise outcomes (range: 6/6–6/38; log MAR 0.0–0.8) compared to the maximum range of control patients (6/6 to 6/12; log MAR 0.0 to 0.3, Levene's F-ratio = 4.52, Figure 3).

Forty-one out of the 60 stroke patients (68%, *p* < 0.0001) showed acquired visual field defects in terms of their Mean Deviation. Fifty percent of patients (i.e., 30/60) had acquired homonymous hemianopias and five (5/60, 8.3%) showed quadrantanopic defects. Three (3/60, 5%) demonstrated altitudinal defects with diffuse loss of visual field sensitivity (Tables 1, 2). The control group showed an average Mean Deviation value of 0.30 ± 0.60 dB whereas the 41 stroke patients who had a significant visual field loss (Figure 3) gave a group Mean Deviation of −5.39 ± 6.26 dB (MWU = 246, *p* < 0.001).

**Table 1:** Vascular territories of lesion sites and corresponding visual deficits.

**Site of Lesion** **R side: *n* = 14** **L side: *n* = 20**	**Total patients (*n* = 34)**	**Hemianopia (*n* = 15)**	**Quadrantanopia (*n* = 1)**	**Altitudinal defect (*n* = 3)**	**VF: MD ± SD (dB)**	**HC VA: mean ± SD (snellen), (mean: log MAR)**	**VA noise: mean ± SD (snellen), (mean: log MAR)**
**Middle Cerebral Artery:** ***n*** **=** **15**							
• R side	7	4	0	1	−6.61 ± 7.29	0.10 ± 0.00,6/7.5	0.34 ± 0.18,6/12
• L side	8	3	1	0	−3.71 ± 4.94	0.10 ± 0.00,6/7.5	0.33 ± 0.15,6/12
**Posterior Cerebral Artery:** ***n*** **=** **6**							
• R side	2	2	0	0	−20.43 ± 0.77	0.10 ± 0.00,6/7.5	0.80 ± 0.00,6/48
• L side	4	4	0	0	−11.56 ± 9.97	0.10 ± 7.79,6/7.5	0.30 ± 0.22,6/12
**Cerebellar Artery:** ***n*** **=** **10**							
• R side	3	1	0	1	−2.01 ± 1.93	0.10 ± 0.00,6/7.5	0.23 ± 0.20,6/9.5
• L side	7	1	0	1	−1.52 ± 3.28	0.10 ± 0.00,6/7.5	0.21 ± 0.15,6/9.5
**Anterior Cerebral Artery:** ***n*** **=** **2**							
• R side	1	0	0	0	−4.23	0.00,6/6	0.10,6/7.5
• L side	1	0	0	0	0.23	0.00,6/6	0.30,6/12
**Internal Carotid Artery:** ***n*** **=** **1**							
• R side	1	0	0	0	−5.83	0.00,6/6	0.10,6/7.5

*(Based on CT/MRI)*.

**Table 2:** Neuro anatomical Lesion from CT/MRI scans and corresponding visual deficits.

**Site of Lesion** **R side: *n* = 12** **L side: *n* = 11 Multiterritorial: *n* = 3**	**Total patients (*n* = 26)**	**Hemianopia (*n* = 14)**	**Quadrantanopia (*n* = 4)**	**Altitudinal defect (*n* = 1)**	**VF: MD ± SD (dB)**	**HC VA: mean ± SD (snellen), (mean: log MAR)**	**VA noise: mean ± SD (snellen), (mean: log MAR)**
**Frontal Lobe:** ***n*** **=** **4**							
• R side	2	1	0	0	−0.61 ± 1.32	0.10 ± 0.00,6/7.5	0.15 ± 0.21,6/9.5
• L side	2	0	1	0	−2.79 ± 3.61	0.10 ± 2.20,6/7.5	0.10 ± 2.67,6/7.5
**Parietal Lobe:** ***n*** **=** **3**							
• R side	1	1	0	0	−7.23	0.10,6/7.5	0·30,6/12
• L side	2	2	0	0	−5.91 ± 6.53	0.10 ± 0.00,6/7.5	0.30 ± 0.40,6/12
**Occipital Lobe:** ***n*** **=** **9**							
• R side	6	5	1	0	−5.05 ± 10.19	0.10 ± 0.00,6/7.5	0.52 ± 0.31,6/15
• L side	3	2	1	0	−6.59 ± 3.81	0.10 ± 0.0,6/7.5	0.40 ± 0.26,6/15
**Pre Frontal Lobe:** ***n*** **=** **1**, R side	1	0	0	0	−0.42	0.10, 6/7.5	0.30, 6/12
**Corona Radiata:** ***n*** **=** **1**, L side	1	0	0	0	−0.07	0.20, 6/9.5	0.50,6/18
**Internal Capsule:** ***n*** **=** **1**, L side	1	0	0	0	−0.24	0.00, 6/6	0.64, 6/24
**Pons:** ***n*** **=** **2**, R side	2	0	0	0	−2.67 ± 3.71	0.05 ± 0.07, 6/7.5	0.25 ± 0.07, 6/9.5
**Basal ganglia:** ***n*** **=** **2**,L side	2	2	0	0	−5.91 ± 6.53	0.10 ± 0.00, 6/7.5	0.30 ± 0.40, 6/12
**Multiterritorial (R & L):** ***n*** **=** **3**	3	1	1	1	−6.10 ± 8.70	0.10 ± 0.17, 6/7.5	0.57 ± 0.32, 6/24

In the right hemisphere, 17/26 and in the left hemisphere 18/31 presented visual field losses in the form of a hemianopia, quadrantanopia or an altitudinal loss. All right hemispheric vascular based lesions showed twice as greater visual field losses compared to left hemisphere (Table 1). Despite the presence of substantial hemianopic and quadrantanopic visual field losses, eighteen of the 41 (44%) patients with visual field loss were unaware of any limitation to their vision. (See Tables 1, 2 for more detailed information on vision function in individual lesion regions.)

The CT and MRI-scans showed that the commonest site of lesion among the 60 patients was a middle-cerebral artery lesion (*n* = 15, 25%), followed by cerebellar artery disorders (*n* = 10, 17%), occipital lobe infarcts (*n* = 9, 15%), posterior cerebral artery lesions (*n* = 6, 10%) and parietal lesions (*n* = 3, 5%). Multi-territorial infarcts were noted in three patients (5%) and the locus for the other 14 cases have been detailed in Tables 1, 2.

## Discussion

To the best of our knowledge, few studies have quantified the incidence and nature of acquired visual deficits in acute ischemic stroke patients (<7 days) with no previous history of visual abnormality (26). The key features of our findings are that most patients (except for 5 cases) with radiologically confirmed first episode ischemic stroke, retain near normal high contrast visual acuity, although given we did not rigorously refract participants but had them wear their habitual reading glasses, we cannot dismiss the possibility that these 5 patients show high contrast visual acuity loss due to uncorrected refractive error. On the other hand, 62% of our patient sample showed deficits in visual acuity-in-noise and 68% showed visual field loss. These changes could not have arisen from an uncorrected refractive state. Besides, although the majority of stroke patients presented with varying clinical symptoms including sudden onset unilateral numbness, loss of motor sensation, and hemiparesis, 44% (18 out of 41) of our patients were unaware of their visual field defect or of their altered visual capacity (i.e., acuity-in-noise).

Our results also demonstrate the clinical potential of using tablet based applications to obtain a quantified measure of visual capacity (visual field, visual acuity and acuity-in-noise) in a relatively short duration (<6 min per eye) in an acute stage of a cerebrovascular injury by testing at the bedside of the patient.

In terms of a structure-function analysis, many ischemic lesions throughout the brain can induce acute visual defects (47). As expected, all occipital lesions (*n* = 9/60) and posterior cerebral artery strokes (*n* = 15/60) induced visual field deficits. All 3 parietal cortex lesions (Right hemisphere: 2, Left hemisphere: 1) also produced visual deficits. Unexpectedly, ~33% (20/60) of cases who had lesions in other regions (Tables 1, 2) of the brain were also associated with visual field deficits and showed an acuity-in-noise impairment. Among them, nine of the 15 middle cerebral artery strokes and four of the 10 cerebellar artery strokes produced visual field defects (Table 1). Two strokes in the left basal ganglia, two out of 4 frontal lobe strokes, and 3 multi-territorial infarcts also caused visual loss (Table 1). The three multi-territorial infarcts involved more than one site of lesion from brain imaging. Interestingly all parietal strokes and the two multi-territorial infarcts which also had parietal lobe involvement produced visual field defects. Although hemineglect is commonly associated with parietal cortex lesions (48), we did not assay for this possibility in the current cohort of patients and cannot comment on its presence.

Our findings are similar to those of Rowe et al. who undertook vision assessment 22 days (median) after stroke (range 0–2,543 days) in patients identified during hospitalization as needing ophthalmic referral. Of these patients 63% had previously shown visual field loss during confrontation test whereas only 37% of cases showed visual field deficits when tested on automated static or manual kinetic (Goldmann) methods (49). From these findings, Rowe et al., concluded that 52% of 915 cases had visual field loss (49). We quantified visual field loss in 68% of our cases who did not have pre-existing eye disease.

Rowe et al. (5) have previously advocated the need for vision testing following stroke. The high prevalence of quantifiable visual defects in acute ischemic stroke cases as noted in our study and that of past works (26), coupled with the lack of awareness for such loss, highlights the need for digital appliances that can quantify these losses. The novel MRFn App is an easy, rapid, and sensitive bedside diagnostic tool for routine use in acute neurological assessments and for tracking recovery or change in the patient.

The immediate impact that acute ischemic stroke *per se* has on visual acuity has not previously been reported even though other neurological diseases such as multiple sclerosis (50) and idiopathic intracranial hypertension (51) are known to be associated with visual acuity loss. Interestingly, 27 out of the 37 patients (73%) who showed deterioration in visual acuity-in-noise also showed evidence of abnormal visual fields but preservation of high contrast acuity.

Clinically, visual acuity is a measure of the ability of the foveal visual system to discriminate a letter or optotype from background spatial information. Visual acuity-in-noise measures the ability to discriminate and identify the targets in the presence of added background white noise (52). The addition of luminance noise imputes to a stronger masking effect for the optotypes, and thus more complex processing of the visual information (31). This is likely the cause of the one line reduction in visual acuity in our controls (mean: 6/7.5-2) in the presence of the noise elements (53, 54). In our stroke group, however, we found a 2-line deterioration in the visual acuity-in noise with mean of 6/12-2. This involves all parietal strokes, occipital strokes, and the multi-territorial strokes with parietal lobe involvement whereas we did not find such a marked visual acuity-in-noise impairment in controls.

The possibility that the visual acuity-in-noise optotypes and visual field loss are measuring similar neuroanatomical processes can be rejected given that patients who showed deterioration in visual acuity-in-noise and visual field sensitivity had regional diversity of lesions (Tables 1, 2) corrupting any commonality in their structure-function relationship (47, 55). Recognition of an acuity target involves the distinction of a static optotype from its background (52). The addition of luminance noise elements raises the threshold of retinal sensitivity as well as the subsequent neural processing needed for stimulus identification (56). This visual processing originates in the primary visual cortex, and involves the dorsal stream via the parietal cortex, for visually guided spatial location and orientation of objects (57). Similarly, ventral processing, which also arises from the primary visual cortex, involves the temporal lobe, and functions in object recognition and the discrimination of object details (58). Thus, it is not surprising that visual acuity-in-noise is affected by stroke as it likely requires processing and possibly integration from extensive cortical regions. The recent work of Cavanaugh et al. (43) in patients who have cortical blindness noted elevated intrinsic noise that affected performance in these patients well-after the acute stroke event (up to 276 months).

It is possible to deduce that the use of visual acuity-in-noise along with high contrast visual acuity at the bedside, has the potential to aid in the diagnosis of ischemic stroke and differentiate these effects from ocular disease. High contrast visual acuity will be typically affected by eye disease and given that visual acuity-in-noise is a sequential processing of this information by cortical inputs through both the dorsal and ventral pathways, these should also be affected due to the reduced ocular input. In our study, our controls returned 0.1 log MAR (6/7.5) for both forms of acuity, whereas stroke cases had an average high contrast acuity of 0.1 log MAR (6/7.5) and an acuity-in-noise of 0.3 log MAR (6/12) implying discrete non-ocular causes for this loss. Patients who had radiologic lesions in their occipital lobes also manifested intact high contrast visual acuity (Table 2).

As 44% of the patients were unaware of their visual field loss, it is also unlikely many would show subjective symptoms of a reduced visual acuity-in-noise as it is a subtle mechanism detected through the testing of target specific features. Although the presence of significant ischemia/brain edema may require longer times (6) for the identification of surrounding objects, we did not place any time constraints on subject response and do not believe that longer observation times would have affected outcomes.

Limitations of our study include the non-identified source of cortical dysfunction, through functional MRI (59), diffusion tensor imaging (60), EEG or psychophysics associated with processes mediated by other cortical regions such as hemispatial neglect (61) or visuomotor processing (62). However, as both of the latter have been reportedly affected by stroke, albeit in a minority of patients, the prospect of loss in cases of generalized cortical involvement is possible. Furthermore, we were unable to identify an association in visual field deficits and visual acuity in noise and hemisphere of lesion. Future studies using functional connectivity (63) MRI may be able to establish this.

Future studies will be required to better establish the mechanisms of functional connectivity associated with cortical defects following acute stroke and during the post stroke recovery phase, especially in visuomotor processing, or attention mechanisms between the right and left side brain hemispheres underlying hemispatial neglect (20) using larger sample sizes for indicators of generalized edema and if, visual acuity-in-noise and some aspect of visual field defects, in the absence of structure-functional relationship, recover over time.

Longitudinal studies with the MRFn app and MRI imaging will elucidate these changes in adaptation, visual attention, and neuroplasticity as well as provide information regarding any therapeutic response in post-stroke patients.

## Conclusion

Our findings indicate that acute stroke induces significant vision loss in 2/3 of hospitalized patients, quantifiable as early as 48-h after stroke, and often unrelated to the confirmed lesion site. Visual acuity-in-noise and visual field deficits have emerged as rapid and sensitive biomarkers of acute ischemic brain dysfunction. Our results imply that visual dysfunction may be associated with a more generalized cerebral dysfunction while highlighting the need for bedside testing of vision for every stroke patient and demonstrating the translational clinical value of the “Melbourne Rapid Field-Neural” iPad application as a low cost, rapid, rigorous and easy to administer functional vision test for use in acute stroke patients.

## Data Availability Statement

The datasets generated for this study are available on request to the corresponding author.

## Ethics Statement

The studies involving human participants were reviewed and approved by Local review board (Western Health Ethics Committee HREC/16/WH/1) and was conducted in accordance with the tenets of the Declaration of Helsinki with all participants (or their carers) providing informed consent. The patients/participants provided their written informed consent to participate in this study.

## Author Contributions

CW was involved in planning, design of the experiments, was responsible for recruitment of patients and all aspects of data collection, contributed to analysis of the data, prepared figures and tables, authored and reviewed the paper, and approved the final draft as part of her doctoral research. TW as Head of Hospital Department of Neurology managed ethical concerns, facilitated patient access and recruitment, was involved in design of experiments, led acquisition and interpretation of all radiological data, contributed to drafting of manuscript, and final approval. AV contributed to design of experiments, led data analysis, preparation of figures and interpretation of visual field results, co-authored and reviewed drafts of the manuscript, and approved the final version. SC conceptualized, designed, funded the study via internal grants, contributed to analysis, theoretical interpretation of the data, drafting of manuscript, and final approval. CW, AV, and SC had full access to all the data in the study. All authors contributed to the article and approved the submitted version.

## Conflict of Interest

AV is a founding director of Glance Optical Pty Ltd, the maker of Melbourne Rapid Field-Neural (MRFn) App. The remaining authors declare that the research was conducted in the absence of any commercial or financial relationships that could be construed as a potential conflict of interest. The handling editor declared a past co-authorship with one of the authors TW.

## Abbreviations

MRFn App: Melbourne Rapid Field Neural App.
